# Epidemiology of sepsis in a university hospital in Rio de Janeiro

**DOI:** 10.1186/cc12963

**Published:** 2013-11-05

**Authors:** Sergio da Cunha, Mario CA Perez, Elisabete N Ferreira, Julio CD Correal, Viviane S Silva, Eliane PP Assumpção, Luana F Almeida, Catherine C Valdez, Jéssica R Oliveira, Thereza CF Camello, Paulo V Damasco

**Affiliations:** 1 Faculdade de Ciências Médicas, Universidade do Estado do Rio de Janeiro, Brazil; 2 Hospital Universitário Pedro Ernesto, Universidade do Estado do Rio de Janeiro, Brazil; 3 Escola de Medicina e Cirurgia, Universidade Federal do Estado do Rio de Janeiro, Brazil

## Background

Severe sepsis and septic shock are challenges in critical medicine care and there are few epidemiologic studies in public university hospitals in Brazil.

## Materials and methods

A prospective study was performed to determine the epidemiology of the sepsis in hospitalized patients in our institution (600-bed tertiary teaching urban hospital) from September 2012 to May 2013. The criteria for sepsis definition were obtained from the 2013 guidelines [1]. Clinical and epidemiological data were collected from patients' records. A univariate, bivariate and multivariate analyses were performed.

## Results

In total, we analyzed 103 patients with severe sepsis and septic shock during the period of study. The frequency of male gender was 55.8% and a median age of 62 years was observed in the patients. The median acute physiology and chronic health disease classification system II (APACHE II) score estimated was 21.1 and a community origin of sepsis was present in 53.4% of them with a mortality rate of 61.3%. Yet in 57 (64.0%) patients with healthcare-associated sepsis, the mortality rate was 63.1% and the risk of death was higher for this group (odds ratio (OR) = 5.54; 95% confidence interval (CI) = 2.19 to 14.0; *P *< 0.05). In the entire group, 53.4% had septic shock and 60.1% entered the vasopressor protocol. In relation to the source of infection, the top three were: pulmonary (51.4%), abdominal (14.5%) and urinary (12.6%). We observed the greatest risk of death in the group with pulmonary infection (OR = 3.08; 95% CI = 1.1 to 8.5; *P *= 0.03). The prevalence of positive blood cultures was 32.1% and 23 microorganisms were identified, these being 65.2% Gram-negative bacilli (*Klebsiella pneumoniae *(21.7%), *Escherichia coli *(17.3%)), 21.7% Gram-positive cocci and 13.6% fungi. Lethality in sepsis episodes was associated independently with the delay in starting antibiotic therapy (more than 6 hours: OR = 2.94; 95% CI = 1.05 to 8.02; *P *= 0.04), inappropriate plasmatic volume expansion use (less than 20 ml/kg: OR = 2.84; 95% CI = 1.07 to 7.5; *P *= 0.03) and pulmonary source of sepsis (OR = 3.08; 95% CI = 1.1 to 8.5; *P *= 0.03). The use of corticosteroids seemed to increase the mortality rate, but in the multivariate analysis this association failed to reach statistical significance (OR = 2.2; 95% CI = 0.08 to 6.5, *P *= 0. 1).

## Conclusions

Enterobacteriaceae and pulmonary sepsis were the main factors responsible for triggering sepsis. Fast and aggressive fluid therapy and early adequate antibiotics are mandatory to change the lethality in severe sepsis and septic shock. Further studies evaluating the effect of therapy with corticosteroids should be assessed.
